# Absence of a sexual dimorphism in postprandial glucose metabolism after administration of a balanced mixed meal in healthy young volunteers

**DOI:** 10.1038/s41387-022-00184-5

**Published:** 2022-02-02

**Authors:** Alessandro Leone, Ramona De Amicis, Simona Bertoli, Angela Spadafranca, Giulia De Carlo, Alberto Battezzati

**Affiliations:** 1 International Center for the Assessment of Nutritional Status (ICANS), Department of Food, Environmental and Nutritional Sciences (DeFENS), University of Milan, Milan, Italy; 2 Istituto Auxologico Italiano, IRCCS, Lab of Nutrition and Obesity Research, Milan, Italy

**Keywords:** Homeostasis, Nutrition

## Abstract

**Background/Objectives:**

A different ability to regulate glucose homeostasis between men and women may contribute to their difference in diabetes prevalence and in its predisposing conditions. Data on this issue are controversial because of heterogeneous protocols and insufficient control of confounders affecting glucose metabolism like age, body composition, and physical activity level. To clarify this issue, we compared among sexes the postprandial glucose metabolism after the administration of a balanced mixed meal normalized to daily energy expenditure.

**Subjects/Methods:**

Thirty-six healthy young volunteers (18 men and 18 women; age, 23.9 ± 2.8 years; BMI, 21.9 ± 1.7 kg/m^2^) were recruited for the experiment. After overnight fast, subjects consumed a mixed meal providing 40% of daily energy expenditure (60% carbohydrates, 25% lipids, 15% proteins) estimated multiplying resting energy expenditure, obtained by Harris & Benedict equation, for the corresponding physical activity level. Blood was sampled at 0, 10, 20, 30, 45, 60, 90, 120, and 180 min and serum concentrations of glucose, insulin, and C-peptide were measured.

**Results:**

Fasting serum glucose concentrations were lower in women than in men, while fasting insulin and C-peptide concentrations did not differ between sexes. Linear mixed models did not show any significant effect of sex and sex # time interaction on postprandial serum glucose, insulin, and C-peptide concentrations. The comparison of areas under the curve between the sexes revealed similar glycemic, insulinemic, and C-peptide postprandial responses between men and women.

**Conclusions:**

Our results do not support the hypothesis of a sexual dimorphism in the regulation of carbohydrate metabolism in young when a mixed meal normalized on individual daily energy expenditure is ingested.

## Introduction

Prevalence of diabetes is globally increasing among all ages [1, 2]. According to recent estimates, the global prevalence of diabetes is higher in men than in women [3]. Moreover, data from several countries also reported that the prevalence of impaired fasting glucose (IFG) and impaired glucose tolerance (IGT) differs by sex, with IFG being more prevalent in men and IGT more prevalent in women [4–8]. It has been suggested that sex may affect the pathophysiology, and thereby the incidence and prevalence, of both type 2 diabetes and conditions preceding its development [9]. In particular, the hypothesis has been put forward that men and women have a different ability to regulate glucose homeostasis. If confirmed, this would have important implications for nutritional strategies for diabetes prevention and management [10, 11].

Data on differences in postprandial glucose metabolism between men and women are controversial, presumably due to the heterogeneity of the approaches used and insufficient control of confounders affecting glucose metabolism. We are aware of one study using an ad libitum caloric intake protocol, where subjects were asked to drink as much as possible of a liquid meal [12]. In this case, the resulted postprandial metabolic response likely depended on the amount of food ingested, which was, in turn, certainly a function of basal hunger level. Other studies have assessed the sex differences in the postprandial glycemic response by administering a mixed meal with a fixed amount of carbohydrates [13] or a mixed meal normalized on body weight [14]. Although valuable, these approaches do not control for important confounders affecting glucose metabolism. It is well-known that, compared to men with same age, women have generally lower skeletal muscle mass and higher adipose tissue mass [15] and this may predispose women to a lower insulin sensitivity and glucose uptake compared to men. On the other hand, women have a higher proportion of body fat in the gluteal-femoral region, whereas men tend to store fat in the abdominal region [16], a known risk factor for insulin resistance and the metabolic syndrome [17]. It is also known that glucose homeostasis is affected by age [18]. Glucose homeostasis may worsen with increasing age, and the time course and magnitude of this deterioration may differ between sexes. Physical activity may be a further a confounder with beneficial effect on insulin sensitivity and glucose uptake [19, 20]. Therefore, it is necessary to control for differences in physical fitness between men and women when assessing sex differences in postprandial glycemic response.

In addition to influencing glucose metabolism, sex, age, body composition, and physical activity level are important determinants of daily energy expenditure. Every day, each individual must consume through food an amount of energy equal to that used by the body to carry out its functions. It follows that the absolute amount of nutrients to be taken in with an experimental meal should not be fixed, but should been varied from person to person depending on their energy expenditure. Thus, for example, energy intake, and the consequent nutrients intake, should be higher in men than in women, in the young than in the elderly, and in those who are physically active than in those with a sedentary lifestyle. A study design involving the administration of a mixed meal normalized to daily energy expenditure would therefore allow to assess the differences between men and women in postprandial glycemic response, controlling for several confounding factors affecting glucose metabolism. Yet, we are not aware of any studies that have used this approach so far.

Therefore, the aim of this study was to investigate the sex differences in glucose metabolism after the administration of a balanced mixed meal, normalized to energy expenditure, in healthy young volunteers.

## Materials and methods

### Subjects

We performed the experiment at the International Center for the Assessment of Nutritional Status (ICANS), University of Milan (Italy), between March and June 2018. Thirty-six (18 women and 18 men) healthy young adults, were recruited on a voluntary basis among students of the University of Milan. Participants were non-smoking men and women, aged 18–35 years, normal weight, and apparently healthy. Subjects were excluded if they were overweight or obese, reported a medical diagnosis of any disease-causing significant impairment of nutritional status (i.e., Crohn’s disease, malignancy, end-stage renal failure, cirrhosis, congestive heart failure, and chronic infection) or endocrine disease (ie, hyper- and hypo-thyroidism and diabetes mellitus), used medications affecting endocrine function in the previous 2 months, had an acute illness or injury in the previous month, or were elite athletes. The use of oral contraceptives was another reason for exclusion from the study. Finally, in order to reduce the effect of sex hormones on glucose metabolism, all women participated in the study during the follicular phase of the menstrual cycle. This study was conducted according to the guidelines laid down in the Declaration of Helsinki. The study was approved by the ethics committee of the University of Milan (protocol n. 32/17). Written informed consent was obtained from all participants.

### Sample selection

Sample size calculation was based on glucose areas under the curves of men and women obtained in a previous study after administration of a mixed meal [14]. With 80% power and a 5% significance level, it was estimated that a sample of 34 volunteers (17 women and 17 men) was sufficient to detect a high effect (Cohen’s *d* = 0.98) in postprandial glucose responses among the sexes.

### Experimental protocol

In the days before the start of the experiment, subjects were invited to our laboratory where a physician performed a detailed medical and clinical examination. Medical history and any drug therapy were recorded. Anthropometric measurements were taken, and abdominal subcutaneous (SAT) and visceral adipose tissue (VAT) thicknesses were measured by ultrasound. A blood sample was also taken in order to exclude subjects with impaired fasting blood glucose (serum glucose ≥ 100 mg/dl) and insulin resistance (HOMA index > 2.5). Subjects were asked to complete the IPAQ questionnaire to assess their level of physical activity [21]. Finally, women were asked to report the first day of their last menstrual period.

We asked subjects to consume, the evening before the experiment, a standardized dinner consisting of pasta or rice seasoned with olive oil and/or parmesan cheese and/or tomato sauce, meat or fish, vegetables seasoned with olive oil, bread, and fresh fruit. In addition, we asked subjects to drink only water.

On the day of testing, subjects arrived at ICANS at 8:30 am fasting. After settling into the room set up for testing, an intravenous catheter was placed in an antecubital vein and a baseline venous blood sample was obtained. Subsequently, the test meal was administered and venous blood samples were obtained at 10, 20, 30, 45, 60, 90, 120, and 180 min after consumption of the test meal to measure serum glucose, insulin, and C-peptide.

### Test meal

The test meal was a balanced meal consisting of a sandwich of white bread, ham, extra virgin olive oil, and tomato. It had to meet 40% of individual daily energy expenditure, obtained by multiplying the resting energy expenditure estimated with the Harris and Benedicts equation [22] by the level of physical activity, using the coefficients proposed by the Italian Society of Human Nutrition (Società Italiana di Nutrizione Umana, SINU) [22]. The meal also had a fixed macronutrient composition (Table 1). Approximately 60% of calories were derived from carbohydrates, 25% from lipids, and 15% from protein. The meal also provided about 9 g fiber per 1000 kcal. Therefore, based on the study design, meal size was different for each individual. Subjects were asked to consume the entire meal within 15 min. During the test, water was always available to participants.

**Table 1:** Energy and macronutrients composition of the test meal.

	Total		Women		Men		*P* value
	Mean	sd	Mean	sd	Mean	sd	
Energy (kcal)	972	125	866	46	1078	78	<0.001
Carbohydrates (g)	151	22	134	11	167	17	<0.001
Carbohydrates (%)	58.1	2.7	58.1	2.7	58.1	2.7	0.584
Proteins (g)	39	5	35	3	42	4	<0.001
Proteins (%)	15.9	1.5	16.1	1.6	15.8	1.4	0.550
Lipids (g)	28	3	25	2	31	2	<0.001
Lipids (%)	25.7	1.3	25.8	1.3	25.7	1.3	0.487
Fiber (g)	9	1	8	1	9	1	<0.001
Fiber (g/1000 kcal)	8.8	1.1	8.9	1.3	8.7	0.9	0.574

The following caloric values were used to convert grams of macronutrients to percent of energy: 3.75 kcal/g for carbohydrates, 4 kcal/g for proteins, and 9 kcal/g for lipids. Abbreviations: *sd* standard deviation.

### Anthropometric measurements

Anthropometric measurements were taken following international guidelines [23]. Subjects were asked to undress, remaining with only the light underwear on, in order to measure body weight and height, waist circumference, and body skinfolds. Weight was measured using a column scale (Seca 700 balance, Seca Corporation, Hanover, MD, USA) to the nearest 100 g. Height was measured using a vertical stadiometer with an accuracy of 0.1 cm. Body mass index was then calculated. Waist circumference was measured with a non-stretch tape midway between the lower rib margin and the superior anterior iliac spine taken to the nearest 0.5 cm. Skinfold thicknesses (biceps, triceps, subscapular and suprailiac) were measured by Holtain Tanner/Whitehouse skinfold calliper (Holtain Ltd, Crymych, Wales). Each skinfold was measured three times and a mean was calculated. Body density and fat mass were then estimated by the Durnin and Womersley equation [24] and by the Siri’s formula [25], respectively.

### Ultrasonography

Abdominal ultrasonography was performed on individuals in fasting state using a Logiq 3 Pro equipped with a 3.5 MHz convex-array probe and with a 7.5 MHz linear probe (GE Healthcare, Milwaukee, WI, USA). VAT and SAT thicknesses were measured 1 cm above the umbilicus. The measurements were taken at the end of expiration and applying a standardized probe pressure. SAT, defined as the distance between the epidermis and the external face of the rectus abdominis muscle, was measured with the 7.5 MHz linear probe; VAT, defined as the distance between the anterior wall of the aorta and the posterior surface of the rectus abdominis muscle, was measured with the 3.5 MHz convex-array probe [26, 27]. Each measurement was taken three times and the mean was calculated.

### Laboratory analysis

Blood glucose and insulin were determined at baseline and at all times mentioned above, and c-peptide was measured at baseline and every thirty minutes up to three hours. All parameters were assayed by commercial kit Roche Diagnostics Italy with Cobas Integra 400 Plus and Cobas 411 (Roche diagnostic, Monza, Italy).

### Statistical analysis

Descriptive variables data are reported as mean ± standard deviation. Two sample comparisons between men and women were made using t-test or rank-sum test for data that were not normally distributed. The effect of sex on continuous outcomes (postprandial glucose, insulin, and c-peptide concentrations) was evaluated using linear mixed models employing the sex (0 = women, 1 = men), time, and a sex # time interaction as fixed-effect predictors and the patient as random effect. A *P* value < 0.05 was considered statistically significant. Statistical analysis was performed using STATA version 12.0 (StataCorp).

## Results

A total of 18 men and 18 women (mean age: 23.9 ± 2.8 years) with normal body weight (mean BMI: 21.9 ± 1.7 kg/m^2^) participated in the present study. Men and women did not differ for age and BMI. As expected, waist circumference was greater in men, whereas the opposite was found for the percentage of body fat. SAT was greater in women, but VAT did not differ between the sexes. Men had a greater daily energy expenditure than women (Table 2).

**Table 2:** Characteristics of the volunteers.

	Total *n* = 36		Women *n* = 18		Men *n* = 18		*P* value
	Mean	sd	Mean	sd	Mean	sd	
Age (years)	23.9	2.8	24.4	3.0	23.4	2.5	0.283
BMI (kg/m^2^)	21.9	1.7	21.8	1.9	21.9	1.5	0.816
Waist circumference (cm)	76.1	4.5	73.3	3.5	78.9	3.6	<0.001
Body fat (%)	20.4	7.7	26.3	5.3	13.9	3.6	<0.001
SAT (mm)	12.0	6.0	14.1	6.9	9.9	4.3	0.030
VAT (mm)	30.9	9.6	29.0	9.6	32.7	9.5	0.260
Resting energy expenditure (kcal)	1577	199	1403	62	1752	114	<0.001
Physical activity (METs/day)	1564	1244	1698	1300	1431	1207	0.475
Daily energy expenditure (kcal)	2454	302	2190	97	2718	177	<0.001

*P* values were obtained using t-test or rank-sum test for data that were not normally distributed. Abbreviations: *sd* standard deviation, *BMI* body mass index, *SAT* subcutaneous adipose tissue, *VAT* visceral adipose tissue.

**Fig. 1:**
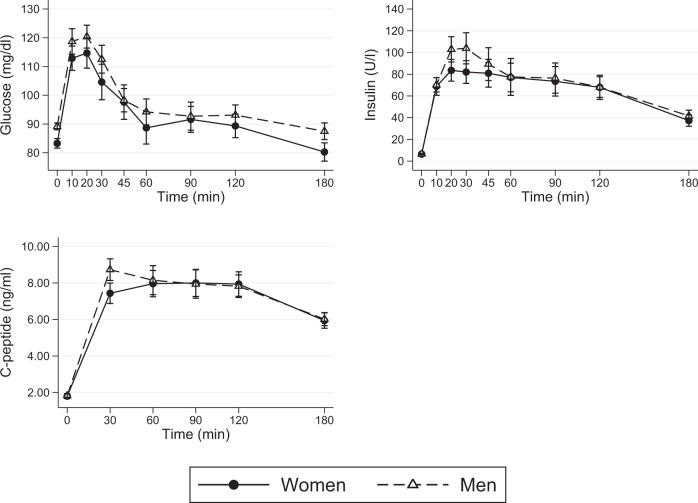
Glucose, insulin, and C-peptide responses to a balanced mixed meal. Values are serum glucose, insulin and C-peptide concentrations, expressed as mean ± standard error, observed in men and women after consumption of a balanced mixed meal.

The glucose, insulin, and C-peptide responses observed in men and women after ingestion of a balanced mixed meal are shown in Fig. 1. At fasting state, men and women differed in glucose concentration (89 ± 5 mg/dl vs. 83 ± 7 mg/dl, *P* = 0.007), but not in insulin (6.6 ± 1.9 U/l vs. 6.5 ± 2.1 U/l, *P* = 0.897) and c-peptide (1.8 ± 0.4 ng/ml vs. 1.8 ± 0.6 ng/ml, *P* = 0.486) concentrations. Applying the linear fixed models (Table 3), we observed no effect of sex and sex # time interaction on postprandial glucose, insulin, and c-peptide concentrations. Only time had a significant effect. In detail, postprandial blood glucose increased by 31 mg/dl (95% CI: 23.81; 38.97) at 20 min, and remained significantly higher than baseline up to 45 min after the meal consumption. Insulin and c-peptide increased by 77.1 U/l and 6.2 ng/ml at 20 and 90 min, respectively, and remained significantly higher than baseline beyond the end of the meal test.

**Table 3:** Linear mixed models showing the effect of sex, time, and sex # time interaction on post-prandial glucose homeostasis.

	Glucose	Insulin	C-peptide
Sex (Men)	5.89	0.09	0.03
	[−5.85,17.63]	[−29.41,29.58]	[−1.55,1.61]
Time (10 min)	29.61^***^	62.29^***^	
	[22.03,37.19]	[43.60,80.97]	
Time (20 min)	31.39^***^	77.07^***^	
	[23.81,38.97]	[58.38,95.75]	
Time (30 min)	21.33^***^	75.54^***^	5.63^***^
	[13.75,28.92]	[56.85,94.22]	[4.74,6.53]
Time (45 min)	14.33^***^	74.39^***^	
	[6.75,21.92]	[55.70,93.08]	
Time (60 min)	5.39	70.52^***^	6.16^***^
	[−2.19,12.97]	[51.83,89.21]	[5.27,7.05]
Time (90 min)	8.33^*^	66.98^***^	6.20^***^
	[0.75,15.92]	[48.29,85.66]	[5.30,7.09]
Time (120 min)	5.90	61.43^***^	6.14^***^
	[−1.81,13.60]	[42.74,80.12]	[5.24,7.03]
Time (180 min)	−3.00	30.81^**^	4.14^***^
	[−10.58,4.58]	[12.12,49.50]	[3.24,5.03]
Male # Time (10 min)	−0.06	1.35	
	[−10.78,10.67]	[−25.07,27.78]	
Male # Time (20 min)	−0.17	19.25	
	[−10.89,10.56]	[−7.18,45.68]	
Male # Time (30 min)	2.06	21.71	1.26
	[−8.67,12.78]	[−4.72,48.14]	[−0.00,2.53]
Male # Time (45 min)	−5.22	8.38	
	[−15.95,5.50]	[−18.05,34.80]	
Male # Time (60 min)	−0.33	0.42	0.16
	[−11.06,10.39]	[−26.01,26.84]	[−1.11,1.42]
Male # Time (90 min)	−4.78	2.86	−0.09
	[−15.50,5.95]	[−23.57,29.29]	[−1.35,1.17]
Male # Time (120 min)	−1.95	0.10	−0.15
	[−12.76,8.86]	[−26.33,26.52]	[−1.41,1.12]
Male # Time (180 min)	1.33	4.25	0.05
	[−9.39,12.06]	[−22.17,30.68]	[−1.21,1.31]
Constant	83.28^***^	6.52	1.80^**^
	[74.98,91.58]	[−14.34,27.37]	[0.69,2.92]

Values are regression coefficients and 95% confidence intervals [in bracket] obtained from mixed-effects linear regression model. Values represent differences in glucose, insulin, and c-peptide concentrations in men compared with women at baseline (effect of sex), changes between a time point and baseline (effect of time), differences in changes between a time point and baseline in men compared to women (effect of sex # time interaction). Abbreviations: *p < 0.05 **p < 0.01 ***p < 0.001

We also tested if the areas under the curve, maximum concentrations achieved, and times to peak of the three outcomes of interest differed between sexes, but we observed no differences between men and women (Table 4). Only insulin tended to peak later in women than in men (58 ± 10 min vs. 29 ± 5 min; *P* = 0.068)

**Table 4:** Hormone and glucose concentrations at baseline and parameters of postprandial glycemic response.

	Total		Women		Men		*P* value
	Mean	sd	Mean	sd	Mean	sd	
Glucose							
Fasting glucose (mg/dl)	86	7	83	7	89	5	0.007
Glucose time to peak (min)	20	10	21	11	19	8	0.621
Glucose max peak (mg/dl)	124	19	120	20	128	18	0.242
Glucose AUC (0–180 min)	10976	7132	10718	7256	11234	7206	0.569
Insulin							
Fasting insulin (U/l)	6.6	2.0	6.5	2.1	6.6	1.9	0.897
Insulin time to peak (min)	44	35	58	41	29	20	0.068
Insulin max peak (U/l)	112.8	63.0	102.2	52.3	123.4	72.1	0.195
Insulin AUC (0–180 min)	12492	7381	12120	7358	12864	7599	0.359
C-peptide							
Fasting C-peptide (ng/ml)	1.8	0.5	1.8	0.6	1.8	0.4	0.486
C-peptide time to peak (min)	71	41	78	37	63	44	0.166
C-peptide max peak (ng/ml)	9.1	3.1	8.8	2.9	9.4	3.3	0.506
C-peptide AUC (0–180 min)	1321	423	1299	437	1343	421	0.477

*P* values were obtained using t-test or rank-sum test for data that were not normally distributed. Abbreviations: *sd* standard deviation, *AUC* area under the curve.

**Fig. 2:**
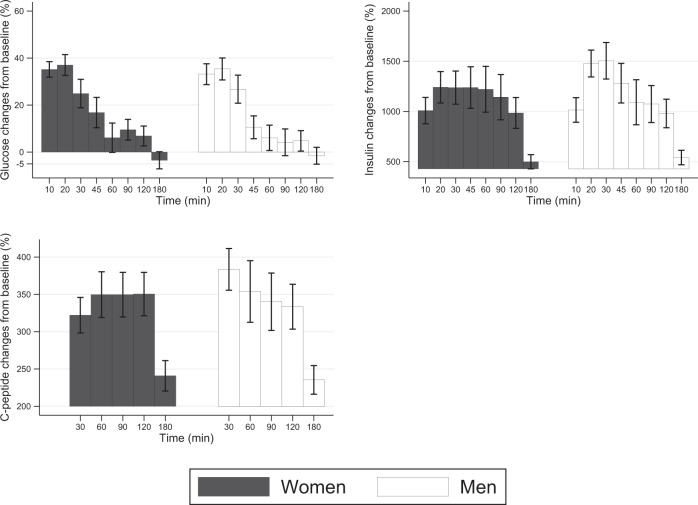
Glucose, insulin, and C-peptide percentage changes in response to a balanced mixed meal. Values are glucose, insulin, and C-peptide percentage changes from baseline (mean ± standard error) observed in men and women after consumption of a balanced mixed meal.

Finally, we explored whether the percentage changes from baseline in serum glucose, insulin, and C-peptide differed between sexes (Fig. 2), but we observed no difference between men and women for all parameters at the times considered.

## Discussion

The present study shows a new approach to study the metabolic response to a mixed meal and provides new results concerning sex differences in postprandial glucose metabolism. Instead of providing an equal amount of carbohydrate between the sexes or an amount normalized on body weight alone, we administered a mixed meal providing a different amount of carbohydrate for each individual based on their daily energy requirements, a parameter that varies between individuals depending on many factors, both genetic and environmental, and which represents the energy required by the body to perform its functions and to maintain a constant body weight. Following this approach, we found that men and women had the same postprandial glycemic increment followed by a similar reduction of glucose concentrations, which returned to baseline values at 2 h from the meal consumption in both sexes. Moreover, our results showed a similar postprandial insulinemic response, tending to peak later in women than in men, and similar C-peptide postprandial blood concentrations between the sexes, suggesting a similar insulin secretion between men and women.

Most previous investigations reported postprandial glucose concentrations to be more elevated in women than in men when a fixed amount of carbohydrates was provided [28–30]. In the AusDiab study, including a large cohort of Australians aged ≥25 years, women had a higher 2 h plasma glucose following an OGTT than in men, but this difference disappeared after adjustment for height [28]. The results were later confirmed by the Inter99 study carried out in a large sample of Danish non-diabetic men and women, where no difference between the sexes in 2 h post-OGTT plasma glucose values was observed when body height was taken into account [29]. This suggests that the higher 2 h post-OGGT glucose level in women may be a consequence of giving a fixed amount of carbohydrate to individuals with different body sizes [28, 29]. The reason for this may be that taller individuals have more skeletal muscle mass for glucose uptake and disposal [28]. Generally, men are taller than women, and for the same height and age, women have less skeletal muscle mass and more fat mass. Thus, women would require more time, compared to men, for glucose disposal when a fixed carbohydrates load is ingested. Also, the higher postprandial insulin and C-peptide concentrations in women following an OGTT [30–32] may to some extent be a consequence of a higher ratio of glucose load per muscular mass in women. As confirmation of these assumptions, in the Australian study [28], authors also reported that men and women had near identical HbA1c values, suggesting similar postprandial glucose excursions in daily life between sexes, and this might be due to the fact that men and women did not eat the same amounts of carbohydrates, but in amounts related to their needs.

Sex differences in postprandial glycemic response were also assessed using an approach in which a mixed meal provided a different amount of carbohydrate between men and women. Basu et al. [14] showed that after ingestion of a mixed meal normalized on body weight (10 kcal/kg) postprandial glucose concentrations were higher in young women than young men. Moreover, postprandial insulin and C-peptide concentrations were higher in young women than in young men, despite the concentrations of these hormone did not differ in fasting state, and the authors suggested that young women had impaired insulin action compared to young men [33]. Several studies using the hyperinsulinemic euglycaemic clamp technique have observed that whole-body insulin-mediated glucose uptake (M) is generally lower in women than in men [31, 34, 35]. However, no sex difference in M was found after correction for body fat [34]. In contrast, when M was normalized per kilogram of muscle mass, insulin action and sensitivity were found to be greater in women as a result of greater glucose disposal [35–37]. This opens up the possibility that the higher postprandial insulin concentration is a consequence of normalizing the meal on body weight rather than on the metabolically more active mass, i.e., muscular mass. A further explanation may be related to the level of physical fitness, an important determinant of insulin action. Authors observed that all measures of fitness and strength were lower in young women than in young men [14], potentially explaining the lower insulin sensitivity in women.

Body composition and physical activity level are determinant of daily energy expenditure. This may explain why when men and women ingest a meal appropriate to their needs, there is no difference in postprandial glucose, insulin, and C-peptide responses. This approach offers several advantages over giving a mixed meal with a fixed carbohydrate load or a mixed meal normalized on body weight. Firstly, this approach allows to control for confounders affecting glucose metabolism, i.e., body composition, age and physical activity level. Secondly, it allows to test the effect of a truly isocaloric physiological meal. Thirdly, the postprandial glycemic excursion achieved with this approach is likely to be more similar to that which occurs in daily life than with other approaches.

The first strength of this study is its innovative approach. We are not aware of any other studies that have investigated sex differences on postprandial glucose metabolism giving a mixed meal normalized on daily energy expenditure. An additional strength of the present study is the narrow age range of the volunteers, a factor that influences glucose homeostasis and whose effect may differ between the sexes. However, this limits the generalizability of the results, which cannot be automatically transferred to other age groups without confirmation by other studies. A further limitation of the study is that the daily energy expenditure was estimated using predictive formulas. However, a recent work comparing data from more than 15,000 indirect calorimetry with resting energy expenditure estimated using the predictive formulae showed good predictive ability and reduced measurement bias from the Harris & Benedict formula in the normal-weight subjects [38]. In addition, by excluding subjects who practiced physical activity at a competitive level, it can be assumed that the bias between measured and predicted energy expenditure is small. An additional limitation is that we did not consider the concentration of sex hormones, which are known to influence glucose metabolism [9]. However, the women participated in the study during the follicular phase of the menstrual cycle, when estrogen levels are low. Finally, gastric emptying, gut hormone responses, pancreatic capacity for hormone secretion, peripheral and central sensitivities to insulin and glucagon, liver weight, and cardiac output could be all relevant sources of variability in postprandial responses, but in this study, they have not been specifically addressed.

In conclusion, our results do not support the hypothesis of a sexual dimorphism in the regulation of carbohydrate metabolism in young. When a balanced mixed meal is ingested, men and women have the same postprandial glucose, insulin, and C-peptide responses. This knowledge may have important implications for strategies to prevent and manage impairments of glucose homeostasis and type 2 diabetes in humans. Further confirmatory studies in elder people are required.

## Data Availability

The dataset analyzed during the current study is available from the corresponding author on reasonable request.
